# Structural, physical, and elastic properties of α-Fe_2_O_3_ nanoparticles doped on borate glasses

**DOI:** 10.1038/s41598-026-40715-z

**Published:** 2026-04-06

**Authors:** Walaa Fouad, S. A. Hussein, M. S. Abd El-sadek, Reham Roshdy

**Affiliations:** 1 Nano & Materials Science Lab, Physics Department, Faculty of Science, Qena University, Qena, 83523 Egypt; 2 Physics Department, Faculty of Science, Galala University, Galala City, Egypt

**Keywords:** Bioactive glass, Nanomaterials, Meltquenching method, Elastic modulus, Medical application, Chemistry, Materials science, Nanoscience and technology, Physics

## Abstract

This study investigates the influence of α-Fe_2_O_3_ nanoparticles (NPs) on the optical, elastic, structural, and physical properties of borate glasses synthesized via the melt-quenching technique. The glass system investigated has the molar composition (68-x) B_2_O_3_-20CaO-2P_2_O_5_-10Na_2_O-xα-Fe_2_O_3_ (x = 0, 0.5, 1, and 2 mol%). Structural analysis using X-ray Diffraction (XRD) confirmed the amorphous nature of all glass sample’s. Fourier-Transform Infrared (FTIR) spectroscopy revealed the presence of structural units containing both three-coordinate (BO_3_) and four-coordinate (BO_4_) boron atoms within the glass network. A clear structural compaction was evidenced by the simultaneous increase in density and decrease in molar volume as the Fe_2_O_3_ content increased. Optically, the indirect optical band gap (Eg) significantly decreased from 3.138 eV to 2.36 eV, concurrently with an increase in the refractive index. This suggests an increase in non-bridging oxygen concentration and enhanced polarizability. Concurrently, elastic measurements revealed a decrease in the elastic moduli with increasing Fe_2_O_3_ concentration, consistent with a weakening or structural rearrangement of the network. Furthermore, several other physical parameters, including packing density, glass dissociation energy per unit volume, optical basicity, polarizability, and average electronegativity, were calculated and discussed as a function of Fe_2_O_3_ concentration, providing comprehensive insight into the structural role of the nanoparticles.

## Introduction

The most important materials in microelectronics, optics, and optical fiber technologies are glasses with high transparency, great chemical durability, and superior thermal, optical, and electrical capabilities^1^. B₂O₃ is one of the best glass formers because of its diverse structural units, low melting point, and strong reactivity and diffusivity with other glass constituents during melt quenching. Pure vitreous B₂O₃ is composed of a random network of BO_3_ triangles and boroxol rings connected by B–O–B linkage.

Iron oxide (Fe_2_O_3_) is a material of considerable scientific and technological interest in glass science, primarily for its role in modifying the optical and structural properties of the final material. Within the amorphous glass matrix, iron exists in an oxidation state equilibrium between divalent (Fe^2+^) and trivalent (Fe^3+^) ions, with these species preferentially occupying distinct structural positions Fe^2+^ typically resides in octahedral sites, while Fe^3+^ favors tetrahedral coordination. This dual-state existence is fundamental to the oxide’s function as a potent coloring agent, where the resultant hues (ranging from yellow to brown) are directly attributable to the specific optical absorption bands associated with each iron species. The integration of iron oxide into a borate glass system, particularly at the nanoscale, establishes a complex structural and chemical interplay, where the iron’s valence state and coordination geometry are highly sensitive to the surrounding glass network composition.

This sensitivity necessitates controlled synthesis, often achieved through precise thermal treatments, to facilitate the formation and evolution of specific iron oxide nano phases, transforming the material into a multi-functional nanocomposite with significant technological potential. These nanocomposites exhibit significantly altered physical properties such as superparamagnetic behavior for magneto-optical applications and enhanced absorption in the UV-Vis region making them valuable for use in electronic devices and switching components^2^. The most stable polymorph, α − Fe_2_O_3_ (hematite), is not only a historical optical colorant but also offers utility in photocatalytic applications due to its stability and low energy gap (≈ 2.1 eV), though the precise mechanism of how doping affects its nanoparticles remains an area requiring further investigation to fully unlock the potential for designing high-performance glass materials.

According to Samdani et al.^3^, who synthesized MgO–BaO–B_2_O_3_– Fe_2_O_3_ glasses, this system has good optical and physical characteristics as well as the ability to shelter photons and neutrons, which makes it appealing for a variety of shielding and optical applications. E. A. Abdel Wahab and et al.^4^ synthesized B_2_O_3_-Pb_3_O_4_-Al_2_O_3_− Fe_2_O_3_ glass system, and examined optical and dielectric characteristics of this system, Consequently, in relation to Fe_2_O_3_ concentration, the basic absorption edge moved towards lower photon energy. We estimate and describe the glass’s absorption coefficient, skin depth, steepness, Urbach tail, and optical band gap. The energy gap dropped from 2.67 to 2.04 eV as a result of this discovery.

Badriah Albarzan and et al.^5^ studied the role of Fe_2_O_3_ doing on the structure and radiation shielding characteristics of 55B₂O₃+30Pb₃O₄+(15-x) Al₂O₃ + x Fe₂O₃ (x = 0, 1, 2, 4, and 5 mol%) glass system. The maximum mass attenuation coefficient (µm) values were obtained at 0.015 MeV and varied from 80.67 to 80.918 cm²/g, respectively, as the Fe₂O₃ concentration increased from 0 to 5 mol%.

This article’s primary goal is to examine how doped α-Fe₂O₃ NPs affect the structure, optical characteristics, and elastic properties of the B₂O₃-Na₂O-CaO-P₂O₅-α-Fe₂O₃ glass system.

## Experimental section

### Glass preparation

**Fig. 1:**
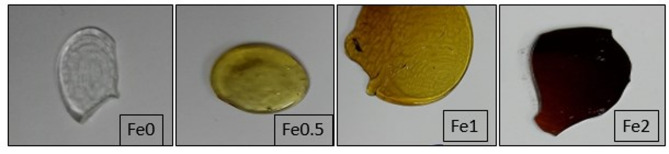
Photograph of the prepared glasses.

The synthesized samples (68- x) B_2_O_3_.20CaO.2P_2_O_5_. 10Na_2_O.x Fe_2_O_3_ (x = 0,0.5, 1, and 2 mol%) were made by melting and quenching method. Table 1 lists the approximate chemical compositions of glasses. Following a thorough weighing of the premium powders of Na_2_CO_3_, NH_4_H_2_PO_4_, CaO, H_2_BO_3_, and α-Fe_2_O_3_ NPs, they were mixed in a grinder. A homogenous, extremely viscous, and transparent liquid was created by heating powders in 50 mL porcelain crucibles to 1000–1050 ^◦^C for one hour in an electric oven. To achieve a high degree of homogeneity, the molten material was constantly stirred. Plate-shaped specimens with the least thickness were produced by rapidly pouring and quenching the molten material between two copper plates that were maintained at a high temperature. Ultimately, a photograph of the samples is shown in Fig. 1. From the picture, Fe_2_O_3_-free sample is colorless whereas the samples containing Fe_2_O_3_ have a brown color that becomes deeper in color with increasing iron content.

**Table 1:** The composition of the synthesis glasses.

α-Fe_2_O_3_	B_2_O_3_	Na_2_O	CaO	P_2_O_5_	Code (mol%)
**0**	**68**	**10**	**20**	**2**	**Fe0**
**0.5**	**67.5**	**10**	**20**	**2**	**Fe0.5**
**1**	**67**	**10**	**20**	**2**	**Fe1**
**2**	**66**	**10**	**20**	**2**	**Fe2**

### α-Fe_2_O_3_ nanoparticles preparation

The precursor material is ferric chloride anhydrous. FeCl_3_ was dissolved in 50 ml of deionized water, then kept under stirring to be a clear solution, and then 30 ml of the extract (6%) was added dropwise to the solution. After that, sodium hydroxide (1 M) was added drop by drop until the pH of the solution reached 2, and the mixture was stirred continuously at 90 °C for 3 h. The separated materials were washed with deionized water 3 times and once with alcohol. The precipitants were dried at (30–45) ^o^C and then calcined at 550 °C for 2 h.

### Sample characterization

The characteristics of the sample were investigated using a variety of methods. The glass nature and α-Fe_2_O_3_ nanoparticles were characterized using an X-ray diffraction (XRD) pattern. Using a copper anode (Cu K, = 1.5406 A) and an X-ray diffractometer (PW 1710 control unit; Philips), it was recorded at room temperature in the 10–80° range. Using a spectrophotometer (Jasco Model 4100 (Japan) infrared spectrometer at ambient temperature and wave-numbers ranging from 400 to 4000 cm^− 1^, the Fourier transform infrared (FTIR) spectra were acquired at 28 ^◦^C using the KBr pellet method. The samples were prepared into a disc shape by mixing them with KBr powder in a 1:100 ratio (sample: KBr, respectively) under an applied load of 100 kg/mm^2^. The mixes were then put through a load of 10 tons/cm^2^ in an evocable die to create transparent, uniform pellets. Using a JASCO 670 UV-Vis spectrophotometer, optical spectra in the 200–800 nm wavelength range were recorded at room temperature.

### Density and molar volume measurements

The densities of the glass samples were measured at room temperature using the Archimedes method using toluene as the immersion liquid with a stable density (ρ_o_ = 0.866 g/cm^3^). The relation that follows illustrates this^6^:

1 $$\:\rho\:=\frac{{W}_{a}}{{W}_{a}-{W}_{t}}{\rho\:}_{o}$$

Where ρ = density of the glass sample,

ρ_o_ = the density of the toluene,

W_a_ = weighing the glass sample in air,

W_t_ = weight in toluene of the glass sample.

2 $$\:\rho\:=\sum\:{D}_{i}{X}_{i}$$

The relationship was used to calculate the molar volume (Vm) values of the glass samples^7^:

3 $$\:{V}_{m}=\frac{\sum\:{X}_{i}{M}_{i}}{\rho\:}$$

Where $\:{M}_{i}$ is the molecular weight of the oxide component (ⅈ), and $\:{X}_{i}$ is the mole fraction.

### The theory of Makishima–Mackenzie

Makishima and Mackenzie used the chemical composition of multi-component oxide glasses to determine the packing density and dissociation energy per unit volume. Where the expression the dissociation energy per unit volume (G_t_) and the packing density (V_t_)^8^:

4 $$\normalsize\:{{V}}_{{t}}=\frac{{\rho\:}}{{M}}\sum\:_{{j}}{{V}}_{{j}}{{X}}_{{j}}$$

5 $$\:{G}_{t}=\sum\:_{j}{G}_{j}{X}_{j}$$

where V_j_ is a packing factor, X_j_ is the mole fraction, and G_j_ is the dissociation energy (kj/cm^3^) of the component oxide j.

To determine the elastic moduli of oxide glasses, Makishima and Mackenzie use the dissociation energy of oxide constituents per unit volume ($\:{{G}}_{{t}})\:$and the packing density ($\:{{V}}_{{t}})$ of chemical compositions^9–11^:

6 $${\text{Young modulus Y}}\,=\,{\mathrm{2}}*{{\mathrm{V}}_{\mathrm{t}}}*{{\mathrm{G}}_{\mathrm{t}}}$$

7 $${\mathrm{Bulk}}{\prime }{\text{s modulus K}}\,=\,{\mathrm{1}}.{\mathrm{2}}*{\mathrm{Y}}*{\text{ }}{{\mathrm{V}}_{\mathrm{t}}}$$

8 $$\:\mathrm{Shear}^{\prime} \mathrm{s}\:\mathrm{modulus}=\frac{3{Y}{K}}{9{K}-{Y}}$$

9 $${\text{Longitudinal modulus L}}\,=\,{\mathrm{K}}+\left( {{\mathrm{4}}/{\mathrm{3}}} \right){\text{ S}}$$

10 $${\text{Poisson ratios}}\,=\,0.{\mathrm{5}} - \left( {{\mathrm{1}}/{\mathrm{7}}.{\text{2 }}{{\mathrm{V}}_{\mathrm{t}}}} \right)$$

Where the Young modulus (Y), bulk modulus (K), shear modulus (S) Longitudinal modulus (L) and Poisson’s ratio (σ).

## Results and discussion

### Spectra of XRD

**Fig. 2:**
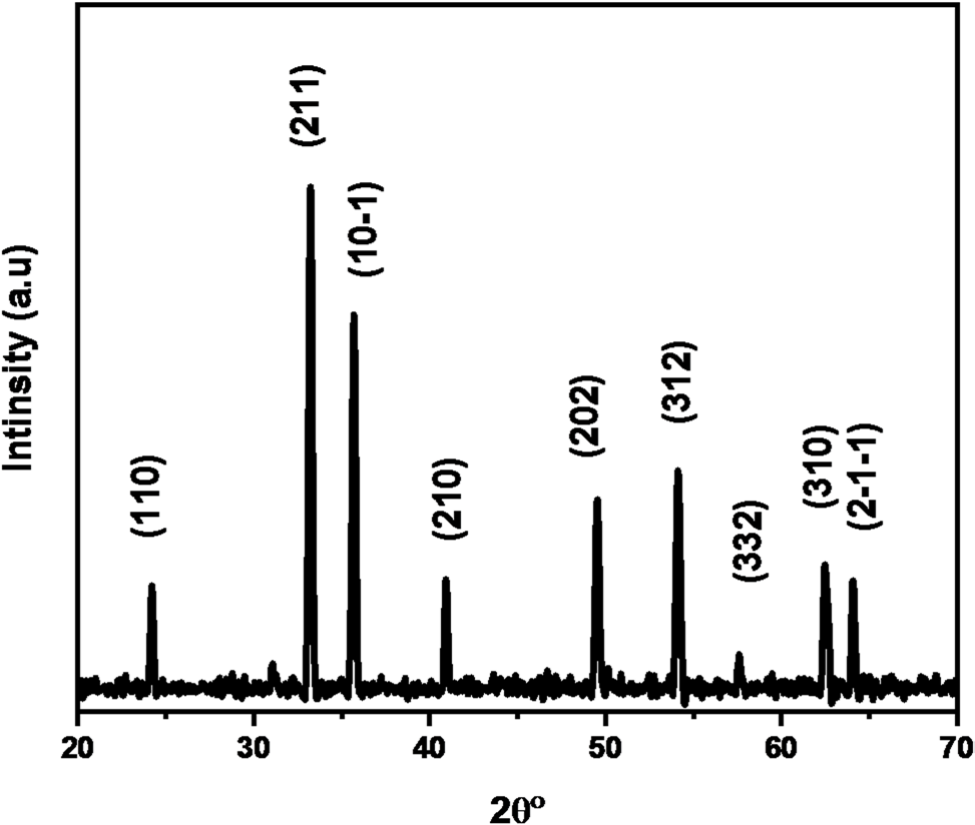
XRD pattern for α-Fe_2_O_3_ NPs.

As shown in Figure 2, it clearly shows the XRD pattern of α-Fe_2_O_3_ NPs. The diffraction peaks were observed at the following (2θ) angles: 24.18°, 33.20°, 35.67°, 40.91°, 49.53°, 54.14°, 57.68°, 62.53°, and 64. 09°. They corresponded to (hkl) planes as (110), (211), (10 − 1), (210), (202), (312), (332), (310), and (2-1-1) planes, and matched with the value on the standard card (JCPDS card No. JCPDS 9009782). The X-ray diffractogram of green synthesized NPs shows the pure α-Fe_2_O_3_ phase of iron oxide with a rhombohedral phase^12^. The crystallite size (D) of these nanoparticles was determined by using the Debye-Scherer formula from the full-width half maximum (FWHM) of the mean peak (211):

11 $$\:D=\frac{K\lambda\:}{\beta\:{cos}\theta\:}$$

K is a constant as a function of crystallite form equal to 0.94, θ is the angle’s Bragg, λ is the X-ray wavelength in nanometers (nm), and β is the full width at half maximum in radians, where the average crystal size (D) of the α-Fe_2_O_3_NPs equals 35.48 nm.

**Fig. 3:**
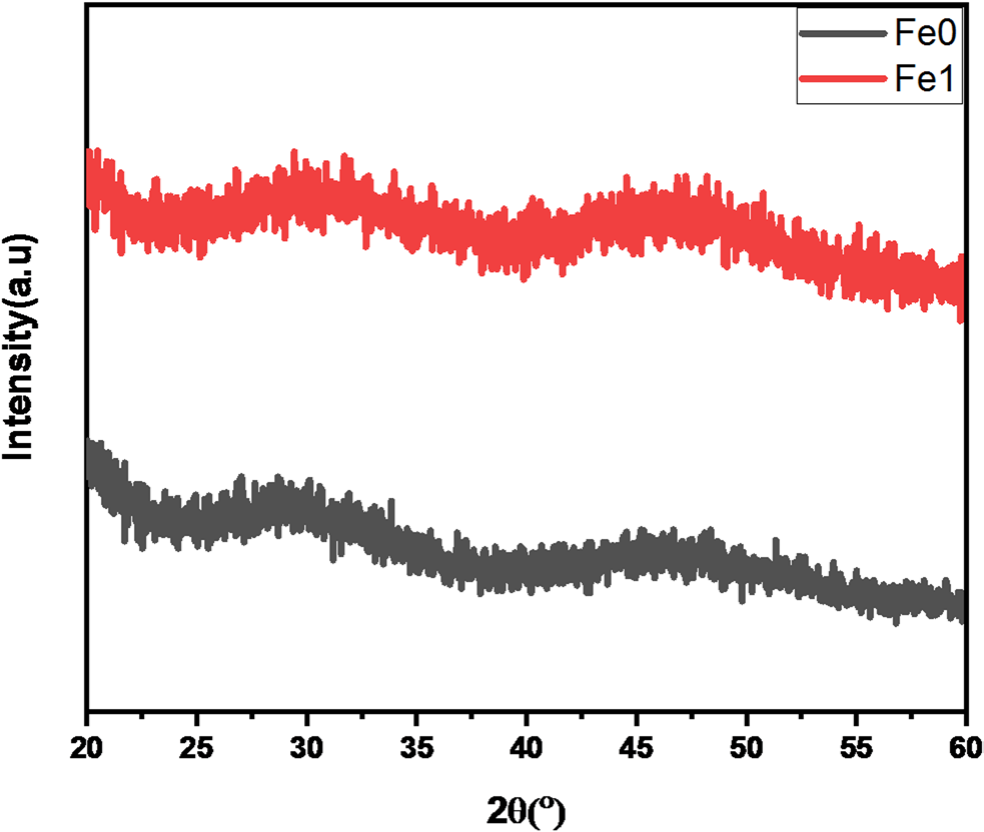
XRD pattern for prepared samples glass.

Examining how X-ray diffraction (XRD) patterns behave through glass samples, as shown in Fig. 3. The absence of diffraction peaks indicate that no crystallizations accrue in the preparing glass and confirms the amorphous nature. The existence of two large humps over 25–35° and 45–50° for 2θ confirms that the sample is amorphous in nature. According to this result, the majority of the Fe₂O₃ did not participate in the glass matrix but instead filled intestinal vacancies as a network modifier as compared to previous work by other researcher^7^.

### Density (ρ) & molar volume (Vm)

**Fig. 4:**
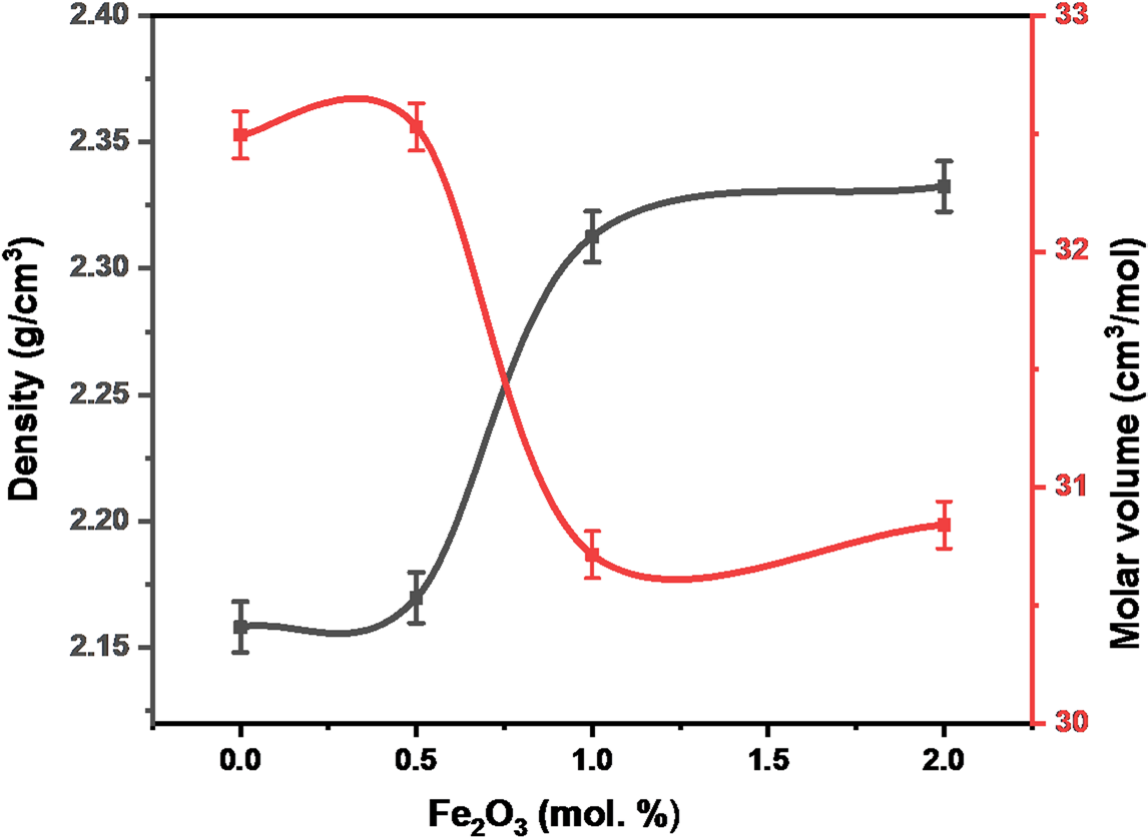
Density and volume molar as a function of α-Fe_2_O_3_ content (error bars: standard deviation: SD, *n* = 3).

The examination of the physical properties of the glass system, as summarized by the data (e.g., Table 6; Fig. 4), reveals a direct correlation between the concentration of α-Fe₂O₃ nanoparticles (x) and fundamental structural metrics and with precision ± 0.01 gcm^− 3^. Specifically, an increase in the molar fraction of α-Fe₂O₃ leads to a notable increase in mass density and a corresponding decrease in molar volume. This increase in density is fundamentally attributed to two factors. First, the systematic substitution of the lighter B_2_O_3_ molecular weight (69.62 g.mol⁻¹) for the significantly larger Fe₂O₃ molecular weight (159.69 g.mol⁻¹) causes a substantial rise in the overall material mass^2^. Second, there is a pronounced density differential between Fe₂O₃ (5.24 g/cm³) and and boron oxide (2.46 g/cm³).

Consequently, the structural units containing the heavier Fe^3+^ ions inherently contribute more to the overall mass density of the glass network than the original B^3+^ units^13,14^. Beyond simple mass substitution, the incorporation of Fe₂O₃ also induces structural changes by supplying additional oxygen to the network. The increase in density could possibly be caused by the creation of additional BO_4_ groups because Fe_2_O_3_ modifies the structural network by introducing more oxygen, which makes it easier for BO_3_ to be converted to BO_4_ units (FTIR section).

### FTIR spectra

**Fig. 5:**
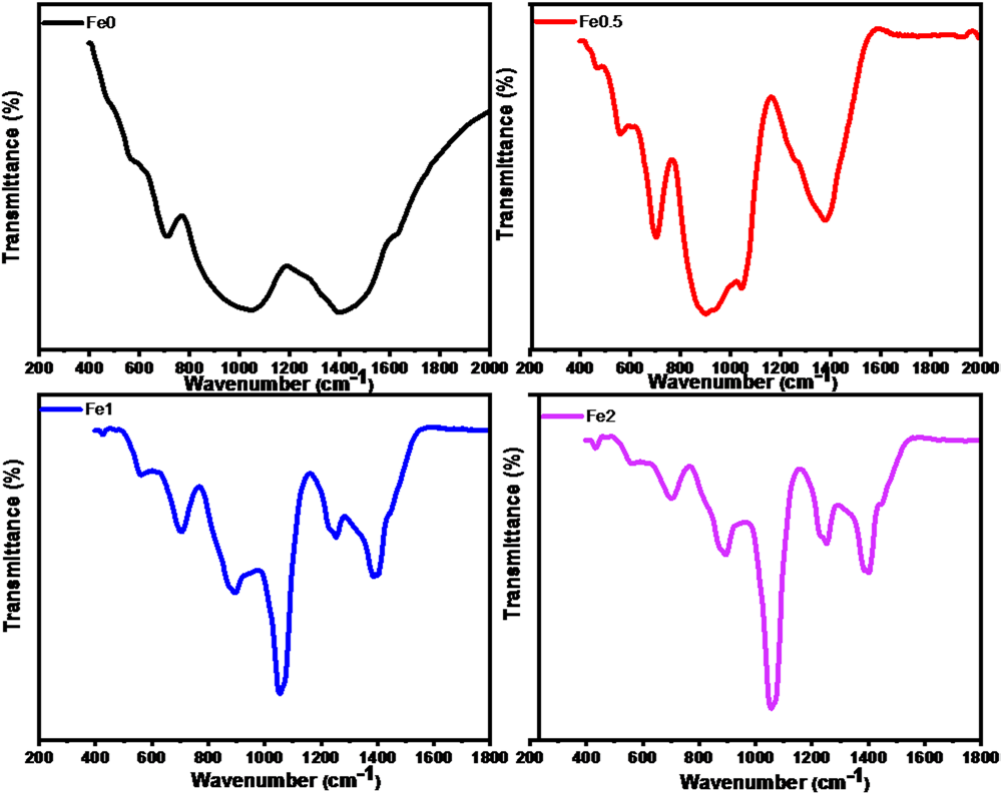
FTIR spectra of prepared glass samples.

B_2_O_3_ glasses are frequently analyzed via a variety of techniques because they are accessible and because their modifier meditation composition has a large number of structural units. The formation of the BO_4_ unit causes the boron coordination to change from 3 to 4 with the introduction of Fe_2_O_3_. The new bond between Fe and (FeO_6_) is the cause of the variation in FT-IR in all samples in the 400–800 cm^− 1^ range, and the strength of the band increases as the amount of Fe_2_O_3_ increases steadily, as illustrated in Fig. 5. The FTIR spectra showed that a broad absorption band at 1384 cm⁻¹ was caused by the B–O stretching vibrations of [BO_3_] units^15^. The symmetric stretching vibration of the O-P-O bond, which is characteristic of pyrophosphate units, is responsible for the peak at 1047 cm⁻¹. The B–O symmetric stretching vibration of BO_4_ tetrahedral units is responsible for the band at 889 cm⁻¹. In symmetric BO_3_ triangles, the absorption band at 699 cm⁻¹ corresponds to B–O–B bending vibrations^16^. The Fe²⁺, Na²⁺, and Ca²⁺ vibrations are associated with the bands 461–554 cm⁻¹.

The FTIR measurement shows that the B-O-B bond’s intensity decreases with increasing Fe_2_O_3_ content, suggesting that the B-O-B bond in the bond ring isolated to BO_3_ units changes into BO_4_ units^17^.

**Fig. 6:**
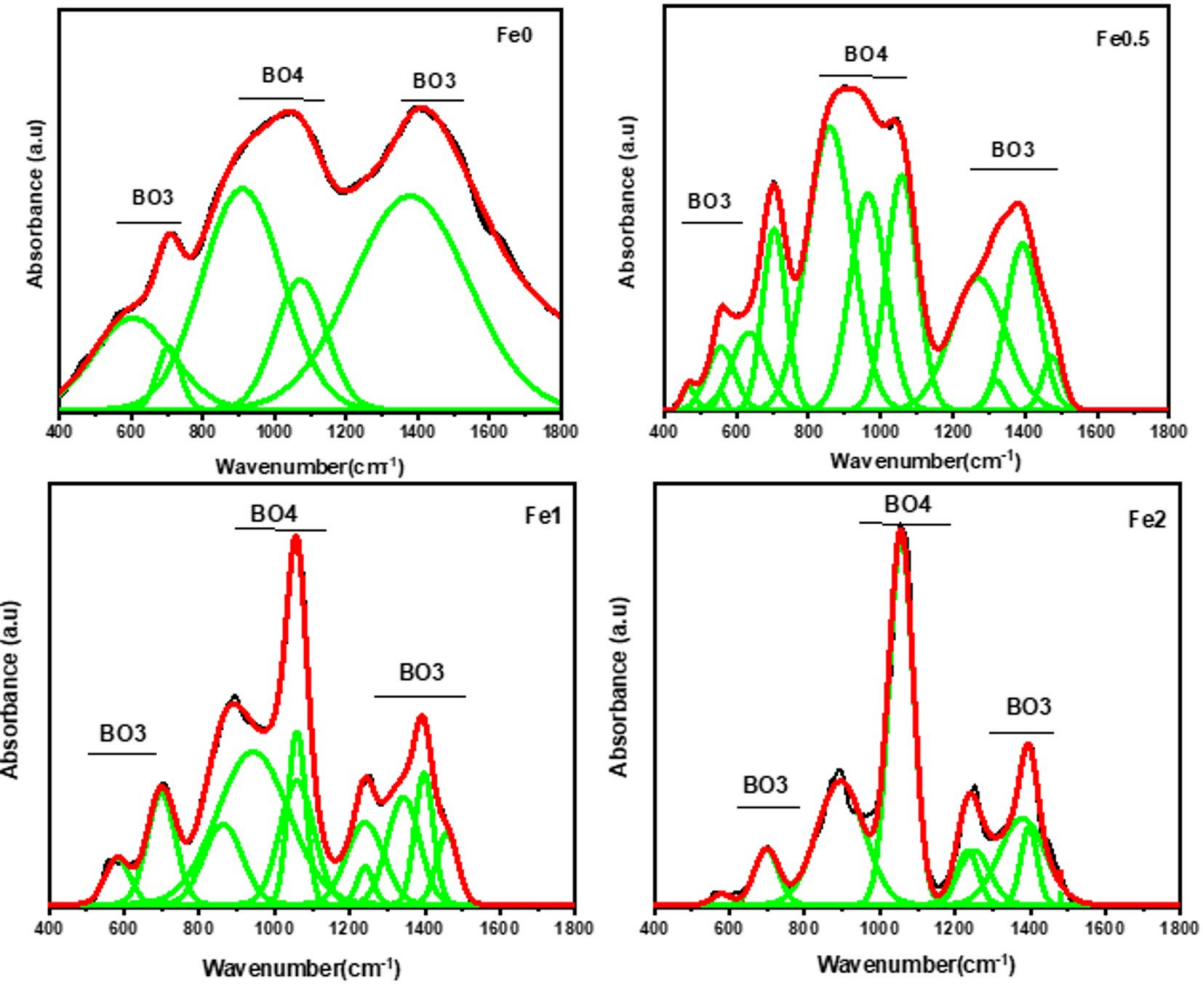
FTIR deconvoluted of samples glass.

Table 2 lists all the peak’s band locations and their assignments. The function group indicating peaks in the FTIR spectra were separated using the deconvolution procedure. Figure 6 displays the FTIR deconvolution of 0, 0.5, 1, and 2 mol% Fe₂O₃ samples as patterns. This figure illustrates that, by promoting the development of B–O bond vibrations of BO_4_ groups, Fe_2_O_3_ functions as a modifier in the present glass system^2,17^. Table 3 including peak centers, FWHM, and relative area percentages from deconvoluted of infrared spectra for glass system.

The N4 was then determined by combining the regions of the BO_3_ and BO_4_ peaks using the formula below:

12 $${\mathrm{N4}}\,=\,{\mathrm{B}}{{\mathrm{O}}_{\mathrm{4}}}/{\text{ }}\left( {{\mathrm{B}}{{\mathrm{O}}_{\mathrm{4}}}+{\mathrm{B}}{{\mathrm{O}}_{\mathrm{3}}}} \right)$$

**Fig. 7:**
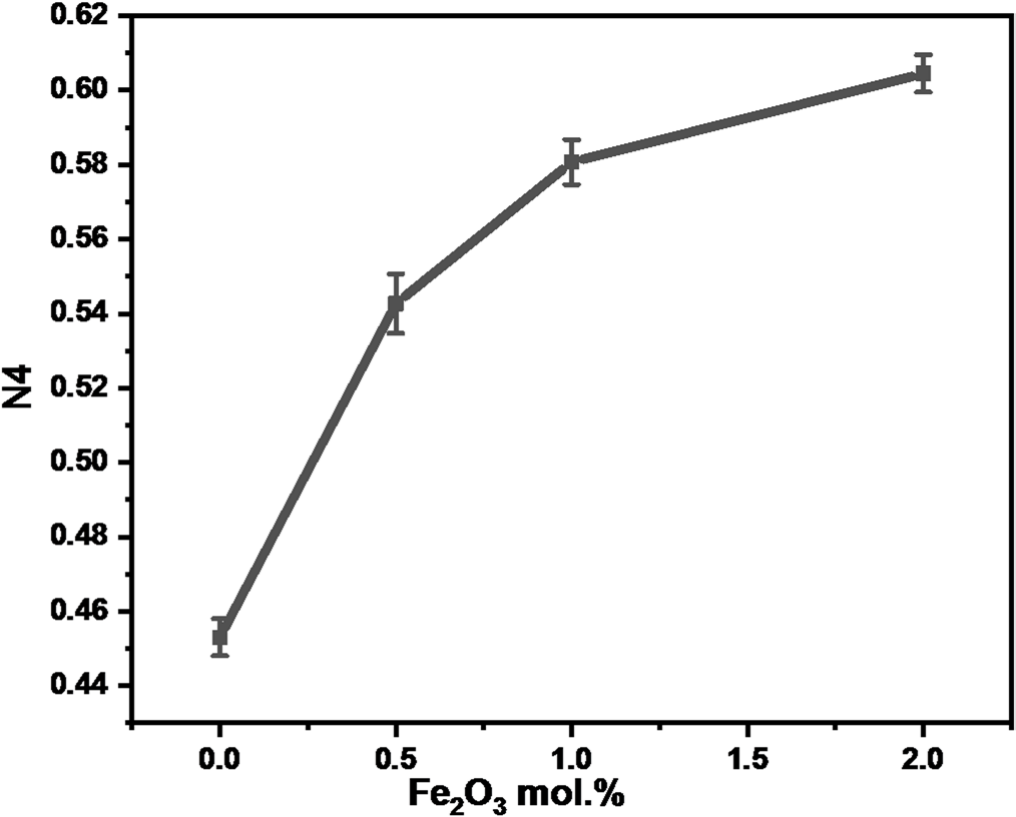
The variation of N4 as a function of α-Fe_2_O_3_ mol%. (error bars: standard deviation: SD, *n* = 3).

Using the correlation between N4 and Fe_2_O_3_ NPs concentration, it was calculated how Fe_2_O_3_ affected the concentration of triangular units (BO_3_) and tetrahedral units (BO_4_). The concentration of Fe_2_O_3_ affected the N4 values, as shown in figure 7. As the concentration of Fe₂O₃ rose, the structural alterations caused by the transformation of the BO₃ into BO₄ structural units led to a rise in N4 levels.

**Table 2:** Band positions (in cm^− 1^) and their assignments of prepared glass samples.

Vibration Groups	Fe0	Fe0.5	Fe1	Fe2
B–O asymmetrical stretching in [BO_3_] unite	1397	1382	1394	1394
B–O stretching vibrations of BO_3_	-	1242	1240	1242
Symmetric stretching vibration of O–P–O bond	1057	1047	1057	1060
B–O symmetric stretching vibration of BO_4_ tetrahedral units	-	870	885	888
B–O–B in BO_3_ units	708	699	703	700
vibrations of Fe^2+^,Na^2+^,Ca^2+^	556	557	558	550

**Table 3:** Center (Xc) **± 5**, area (A) **± 1** and FWHM (W) **± 5** obtained from deconvoluted of infrared spectra for glass system.

Peak. No	Fe0					Fe0.5				Fe1				Fe2			
1	Xc		A		W	Xc		A	W	Xc		A	W	Xc		A	W
2	1378.22		179.77		381.6	1475.1		0.73	66.93	1458.7		1.38	66.33	1480.77		0.12	0.86
3	1072.3		48.47		169.51	1392.52		3.62	107.61	1397.52		2.22	58.04	1396.42		1.73	51.31
4	909.88		128.78		264.64	1320.33		0.33	55.62	1342.84		3.15	100.85	1378.68		5.39	143.07
5	704.297		10.66		76.45	1268.94		4.77	178.37	1240.75		2.78	117.05	1250.83		2.35	100.03
6	604.56		52.48		259.27	1058.08		4.83	101.89	1240.75		0.55	50.13	1232.98		1.55	65.66
7	−		−		−	964.54		5.14	117.59	1059.12		3.84	107.17	1056.33		12.31	76.45
8	−		−		−	857.91		8.52	148.62	1059.12		2.78	55.36	894.93		7.95	147.86
9	−		−		−	704.9		2.82	77.06	942.642		9.98	224.49	875.15		0.047	0.07
10	−		−		−	635.35		1.83	116.85	862.5		2.82	120.76	697.88		1.844	76.52
11	−		−		−	556.28		0.11	30.24	698.22		2.873	88.59	579.41		0.29	60.96
12	−		−		−	556.28		1.17	91.6	581.6		1.061	78.85	−		−	−
13	−		−		−	467.19		0.208	44.62	−		−	−	−		−	−

### Mechanical properties of glass system (68-x) B₂O₃.20CaO.2P₂O₅. 10Na₂O.xFe₂O₃ system

**Fig. 8:**
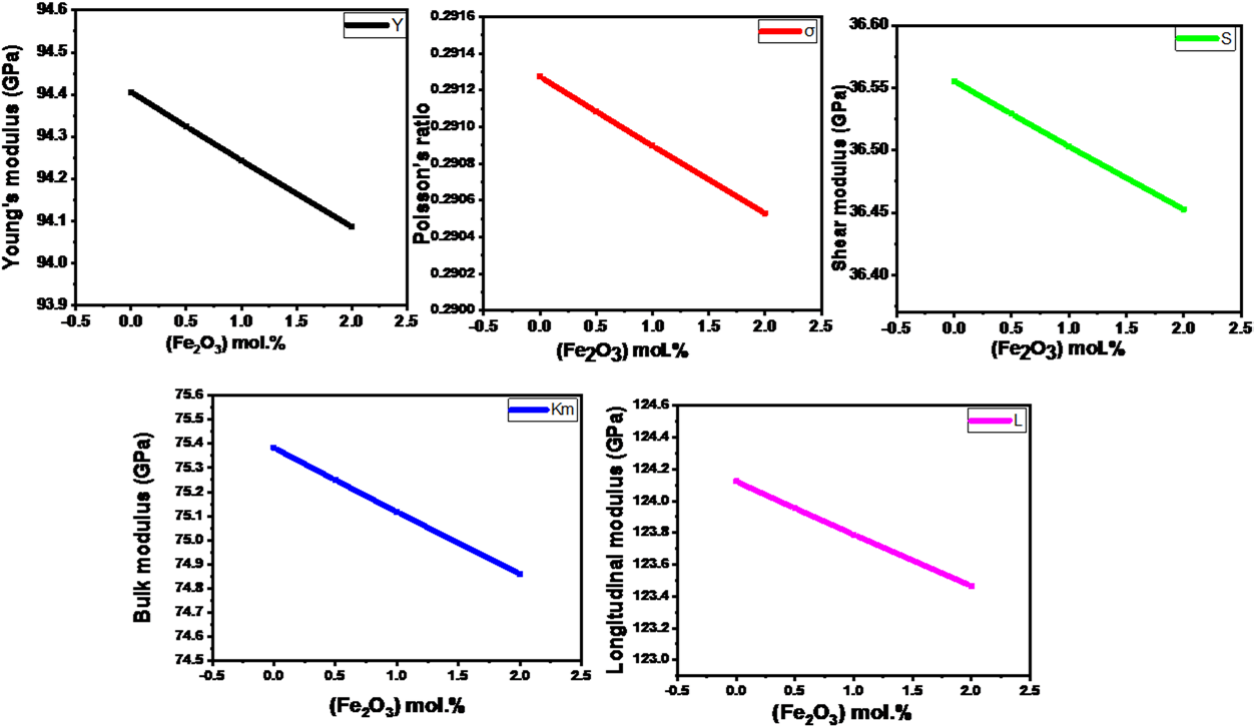
Elastic’s modulus as a function of α-Fe_2_O_3_ content.

In the base glass, the strong network is formed by B_2_O_3_ (BO_3_ and BO_4_ units) and P_2_O_5_ (PO_4_). When Fe₂O₃ is introduced at the expense of B_2_O_3_, the Fe^3+^ ions tend to act as network modifiers at these low concentrations, despite their potential to act as formers (like FeO_4_) in other compositions. The Makishima–Mackenzie mode is used to compute the molecular weight (M_i_), packing density (V_t_), Young’s modulus (Y), Poisson ratio (σ), shear modulus (S), bulk modulus (K) and Longitudinal modulus (L**)**, tabulated in Table 4 of [(68-x) B_2_O_3_−20 CaO-2P_2_O_5_−10Na_2_O-x (Fe_2_O_3_)] glasses system (x = 0, 0.5, 1, and 2 mol%; with names Fe0, Fe0.5, Fe1, and Fe2, respectively). The elastic moduli of the glasses begin to decrease as α-Fe₂O₃ content rises, as seen in Fig. 8. The findings show that the elastic moduli of the glasses begin to decrease as the Fe₂O₃ content rises. It was discovered that variations in the samples’ elastic properties responded to packing density changes in similar manners. The elastic modulus decreased when Fe₂O₃ was added because it raised the glass sample’s ionic radii, where the ionic radii of the Fe^3+^ (0.92 Å) are greater than those of the borate ion (0.351 Å)^11^. Table 5 contains the Oxide Density (ρ), Dissociation Energy (Gj), and Packing Factor (Vj).

When a material is subjected to a straining force, the Poisson ratio is the ratio of its axial or longitudinal strain to its transverse or lateral strain. Furthermore, the values of Poisson’s ratio (σ) have a decreasing behavior with more Fe_2_O_3_ additives. The reduced behavior of σ indicates that the lateral strain will be less than the longitudinal strain if samples are subjected to the same load. Additionally, Y can be used to compute bulk modulus (K) and shear modulus (S) values. This results shown that for borate glasses with additional Fe_2_O_3_ additives, the values of S and Km decreased. This decline is a clear manifestation of network depolymerization, structurally confirming that the Fe^3+^ ions act predominantly as network modifiers at these low substitution levels, despite iron’s potential dual role in glass formation. Mechanistically, the incorporation of Fe_2_O_3_ disrupts the strong, covalently bonded network (B-O-B and P-O-P linkages), preferentially breaking these non-bridging bonds to create a larger fraction of bridging oxygen (NBO) species. The resulting looser atomic structure requires less stress for a given strain, thus lowering the Young’s modulus. Furthermore, the modifying action of the Fe^3+^ ions, competing for available oxygen, contributes to a reversal of the strengthening effect of the boron anomaly, converting some stable four-coordinated boron (BO_4_) units back to the weaker, less-connected three-coordinated (BO_3_) units. This structural shift, often correlated with a concurrent increase in Poisson’s ratio (σ) unequivocally indicates a reduced mechanical stability within this compositional range.

**Table 4:** Molecular weight (Mi), packing density (Vt), and elastic moduli (Y, Km, S,Land σ) calculated according to Makishima–Mackenzie mode of glasses.

Sample	M_i_(g/mol.)	V_t_(cm^3^/mol.)	Y(GPa)	S(GPa)	σ(GPa)	Km(GPa)	L(GPa)
Fe0	70.13	0.665	94.406	36.56	0.2913	75.38	124.12
Fe0.5	70.58	0.6648	94.32	36.53	0.2911	75.25	123.95
Fe1	71.03	0.664	94.245	36.5	0.291	75.12	123.79
Fe2	71.94	0.663	94.09	36.45	0.2905	74.86	123.46

**Table 5:** Density(ρ), Dissociation Energy (Gj) and Packing factor (Vj) of Oxides.

Oxide	ρ (g/cm^3^)	Vj (cm^3^/mol.)	Gj (kcal/cm^3^)	Gj (kJ/cm^3^)
B_2_O_3_	2.46	20.8	18.62	77.9
Fe _2_O_3_	5.24	21.6	18.76	78.5
Na_2_O	2.27	11.2	8.9	37.3
CaO	3.34	9.4	15.51	64.9
P_2_O_5_	2.39	34.8	15	62.8

### Properties of optical

**Fig. 9:**
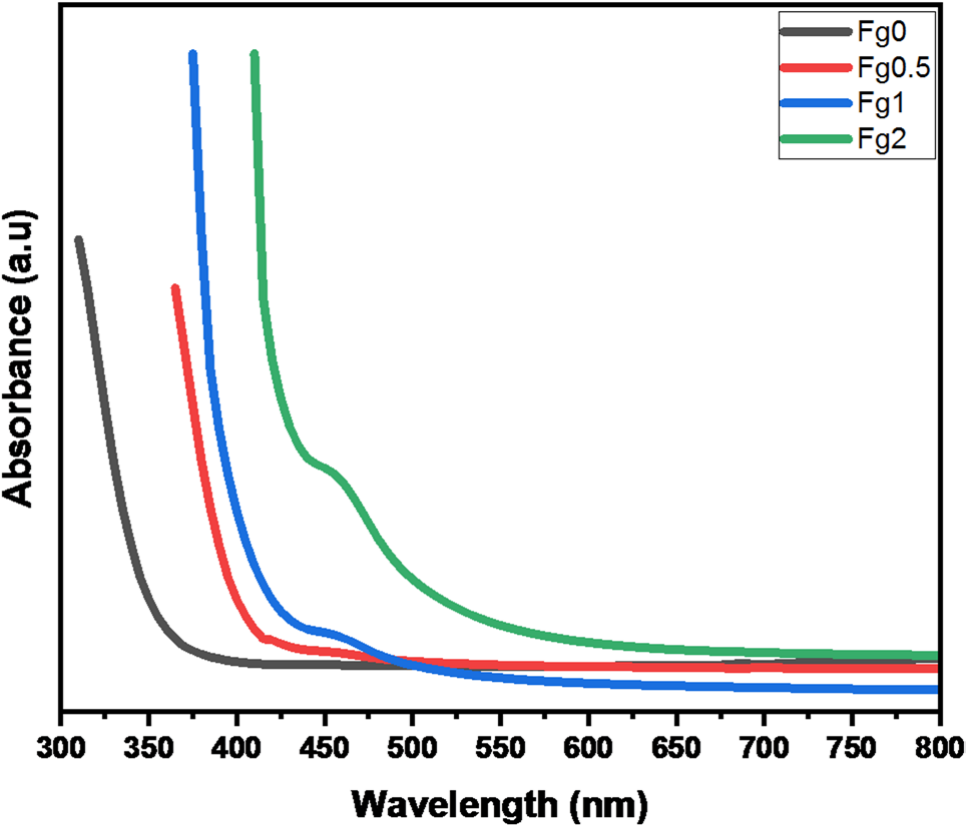
Absorption spectra of the prepared glass as a function of wavelength.

The optical absorption spectra for [(68-x) B_2_O_3_−20 CaO-2P_2_O_5_−10Na_2_O-x Fe_2_O_3_] (x = 0, 1, 2, and 3 mol%; with names Fe0, Fe0.5, Fe1, and Fe2 respectively) glasses was presented in Fig. 9. There are absent strong peaks in the optical absorption spectra. It is evident that the basic absorption shifts to a higher wavelength (low energy) as more Fe₂O₃ is introduced to the glass system. This happens as a result of the studied glasses’ optical band gap being reduced, which improved the electronic transitions’ capability. Only one absorption band, located at approximately 458 nm, has been detected in the UV-visible optical absorption spectra of the glasses under study. This band results from Fe³ electronic transitions^18^. On the other hand, Fe_2_O_3_ doped samples have a brown color. Moreover, with increasing Fe_2_O_3_ content, the color becomes darker; the brown color is an indication that the Fe has a trivalent oxidation state^14^.

The Tauc equation suggests a relationship between absorption coefficients and photon energy:

13 $$\alpha {\mathrm{h}}\nu \,=\,{\mathrm{A}}{({\mathrm{h}}\nu - {\text{ }}{{\mathrm{E}}_{\mathrm{g}}})^{\mathrm{n}}}$$

**Fig. 10:**
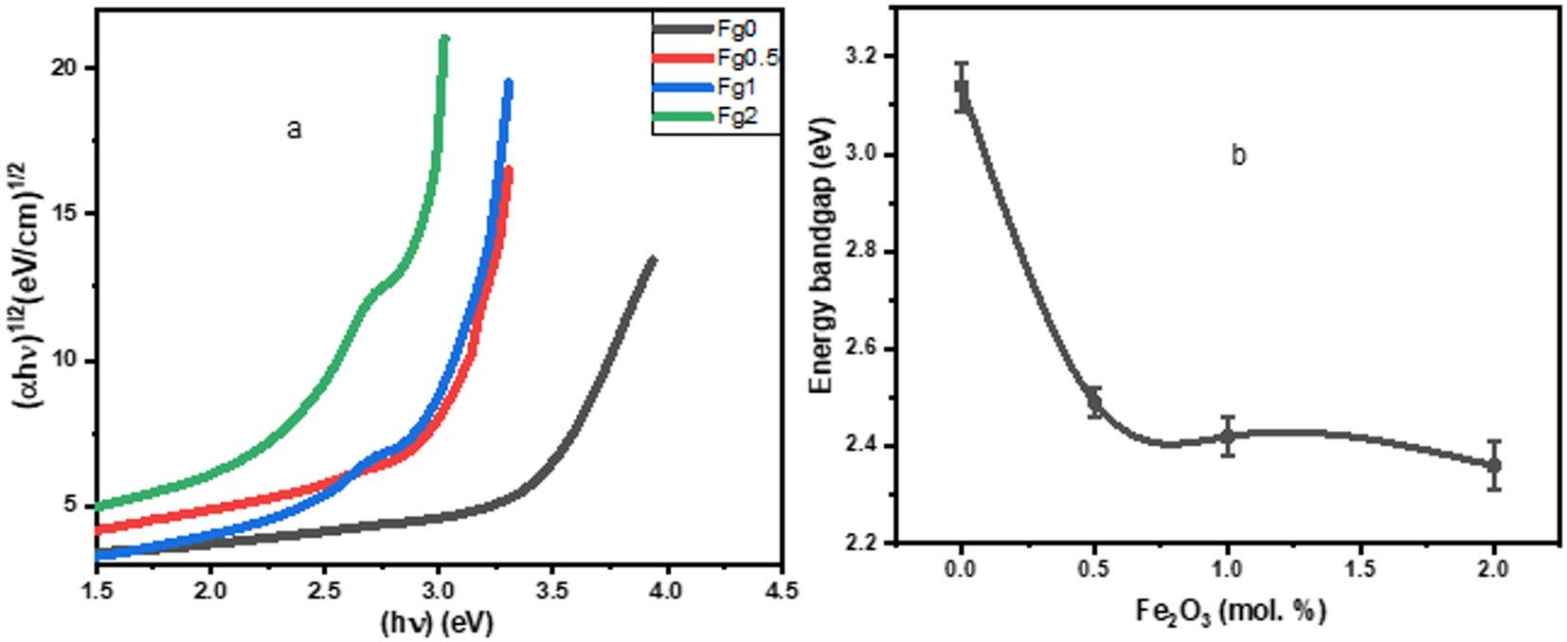
(a) Tauc’s plots for the present glass system and (b) bandgap values versus Fe_2_O_3_ content (error bars: standard deviation: SD, *n* = 3).

In this context, A represents a constant, (α) denotes the optical absorption coefficient, (hv) signifies photon energy, and n indicates the type of transition, where n is ½ for direct allowed transitions and 2 for indirect transitions. Figure 10a shows the Tauc plots for present samples. The optical band gap is dependent on how the glass’s structure changes following the addition of a modifier. The results showed that Eg decreases when Fe_2_O_3_ ions were added, as shown in Fig. 10b. The discrepancy between the broad energy gap of B₂O₃ (~ 6.20 eV) and the energy gap of α-Fe₂O₃ (~ 2.1 eV) could be the cause of the optical band gap’s lowering^4,19^. The absorption coefficient (α) was calculated using the relation α = 2.303*(A/d), where A is the absorbance and d is the thickness of the sample. The thickness value of samples (0.23,0.13,0.14 and 0.16 cm) respectively was determined via [Vernier Caliper]. The refractive index (n) from E_g_ obtained from the UV-Vis data is calculated using the formula below:

14 $$\:{\mathrm{n}}^{2}=\sqrt{\frac{180}{\mathrm{E}\mathrm{g}}}-2$$

**Fig. 11:**
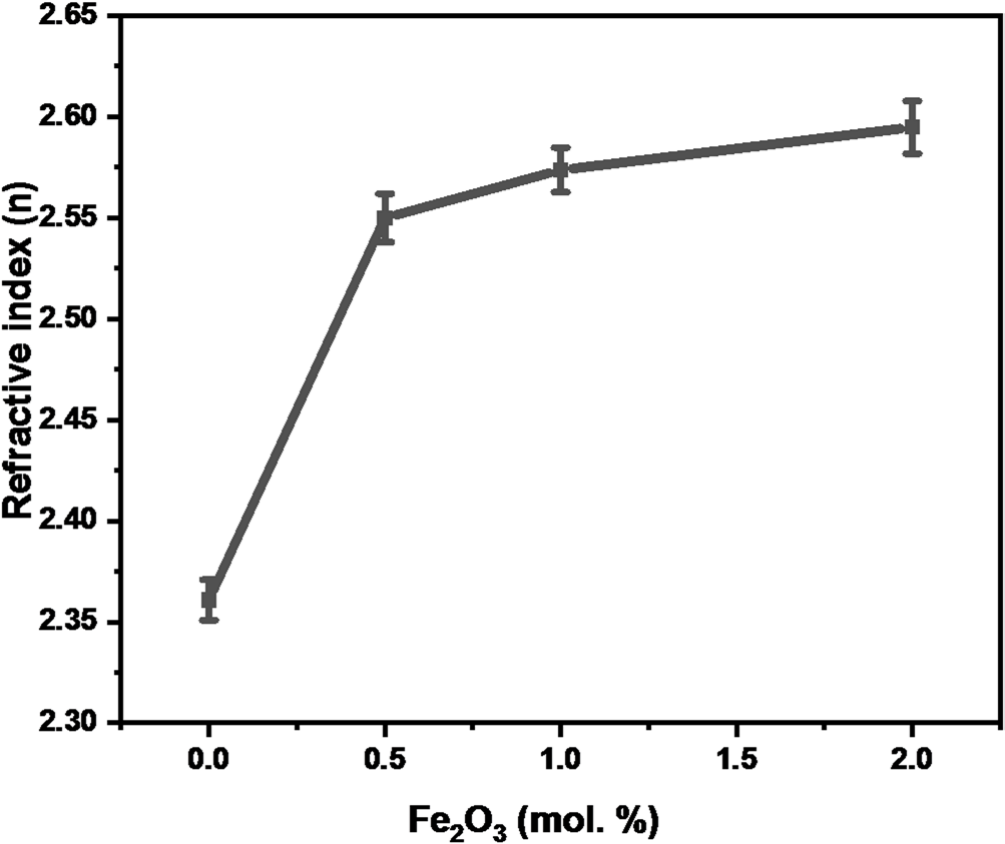
Refractive index of the prepared glass (error bars: standard deviation: SD, *n* = 3).

The glasses under study refractive index is displayed in Fig. 11. Thus, the increased density and polarizability of the glass are responsible for the rise in refractive index values^20,21^. As the wavelength and the amount of Fe₂O₃ in the glasses under study grow, so does their refractive index. The following formula can be used to determine metallization (M):

15 $$\:\mathrm{M}=\sqrt{\frac{\mathrm{E}\mathrm{g}}{20}}$$

The results indicate that the M values vary between 0.39 and 0.34, as tabulated in Table 6, so they are decreasing, reflecting the semiconductor nature of the samples. Materials having a M value between 0.34 and 0.45 have been shown by Dimitrov and Komatsu to have a significant non-linear refractive index, which could make them valuable as novel nonlinear optical materials^16,22^.

The overall polarizability of a mole of a material is measured by molar refraction. The Lorentz-Lorenz relationship relates to isotropic substances such as liquids and glasses.it is related to the structure of the glass. So, the molar refractivity Rm (cm^3^) of samples is calculated from the equation^23^:

16 $$\:\mathrm{R}\mathrm{m}=\mathrm{V}\mathrm{m}(1-\sqrt{\frac{\mathrm{E}\mathrm{g}}{20}}\:)$$

The term electronic polarizability refers to the extent to which electrons respond to an electric field. A material’s molar refraction (Rm) and molar electronic polarizability (α_m_) are proportional according to the Clausius–Mosotti connection^4^:

17 $$\:{{\upalpha\:}}_{\mathrm{m}}=\mathrm{R}\mathrm{m}\:\left(\frac{3}{4\mathrm{N}{\uppi\:}}\right)$$

Avogadro’s number is denoted by N. The equation becomes the following equation when $\:{{\upalpha\:}}_{\mathrm{m}}$ is added to (Å^3^)^24^:

18 $$\:{{\upalpha\:}}_{\mathrm{m}}=\left(\frac{\mathrm{R}\mathrm{m}}{\mathrm{2,52}}\right)$$

Table 6 contains the values for electronic polarizability and molar refraction^22^. Consequently, an increase in the Fe₂O₃ content raises the refractive index and increases the polarizability of oxide ions. This behavior is explained by an increase in molar refraction, which raises the measured refractive indices when ion oxide’s electrical polarizability increases^25^.

Each glass’s refractive index value (ε = n^2^) was used to record the dielectric constants (ε), and the following relation was used to record the samples’ optical dielectric constant:

19 $$\:{\mathrm{P}}_{\mathrm{d}\mathrm{P}}^{\mathrm{d}\mathrm{t}}={\upepsilon\:}-1={\mathrm{n}}^{2}-1$$

Table 6 shows that the dielectric and optical dielectric constants in the glass sample increased as the dopant (Fe_2_O_3_) concentration increased. The dielectric constant usually rises with increased polarizability from the electronic and ionic contributions^26^.

### Optical basicity, polarizability, and average electronegativity

The optical basicity of the glasses was used to provide information about their acid-base characteristics, and it is linked to the electron density carried by oxygen. Moreover, it describes the ability of oxygen negative ions within the glass matrix to transfer electron to positive ions^13^. Additionally, the following formula is used to calculate ᴧ_th_ using the Duffy and Ingram method:

20 $$\:{ \Lambda }_{\mathrm{t}\mathrm{h}}\:=\frac{0.75}{{{\upchi\:}}_{\mathrm{a}\mathrm{v}}-1.35}$$

Where the electronegativity, represented by $\:{{\upchi\:}}_{\mathrm{a}\mathrm{v}}$, can be shown using the following equation:

21 $$\:{{\upchi\:}}_{\mathrm{a}\mathrm{v}}=\sum\:{{\upchi\:}}_{\mathrm{i}}{\mathrm{x}}_{\mathrm{i}}$$

Where, using Pauling’s scale, $\:{\mathrm{x}}_{\mathrm{i}}$ is a cation’s electronegativity and $\:{{\upchi\:}}_{\mathrm{i}}$is its concentration %. As the quantities of Fe_2_O_3_ increase, the glass network tends to become more ionic and less covalent, as indicated by the increased electronegativity values.

The properties of several materials, such as ferroelectricity, conductivity, refraction, electro-optical effect, optical basicity, and optical nonlinearity, are closely related to electronic polarisability. Optical basicity, which is strongly influenced by electrical polarisability, has also been shown to be an essential and necessary prerequisite for predicting the properties of a glass system before the glass is used in different applications^13^.

It is possible to determine the electrical polarisability of oxide ions ($\:{{\upalpha\:}}_{0}^{-2})$ using the following formula:

22 $$\:{{\upalpha\:}}_{0}^{-2}=\frac{1.67}{1.67-{ \Lambda }_{\mathrm{t}\mathrm{h}}}$$

**Fig. 12:**
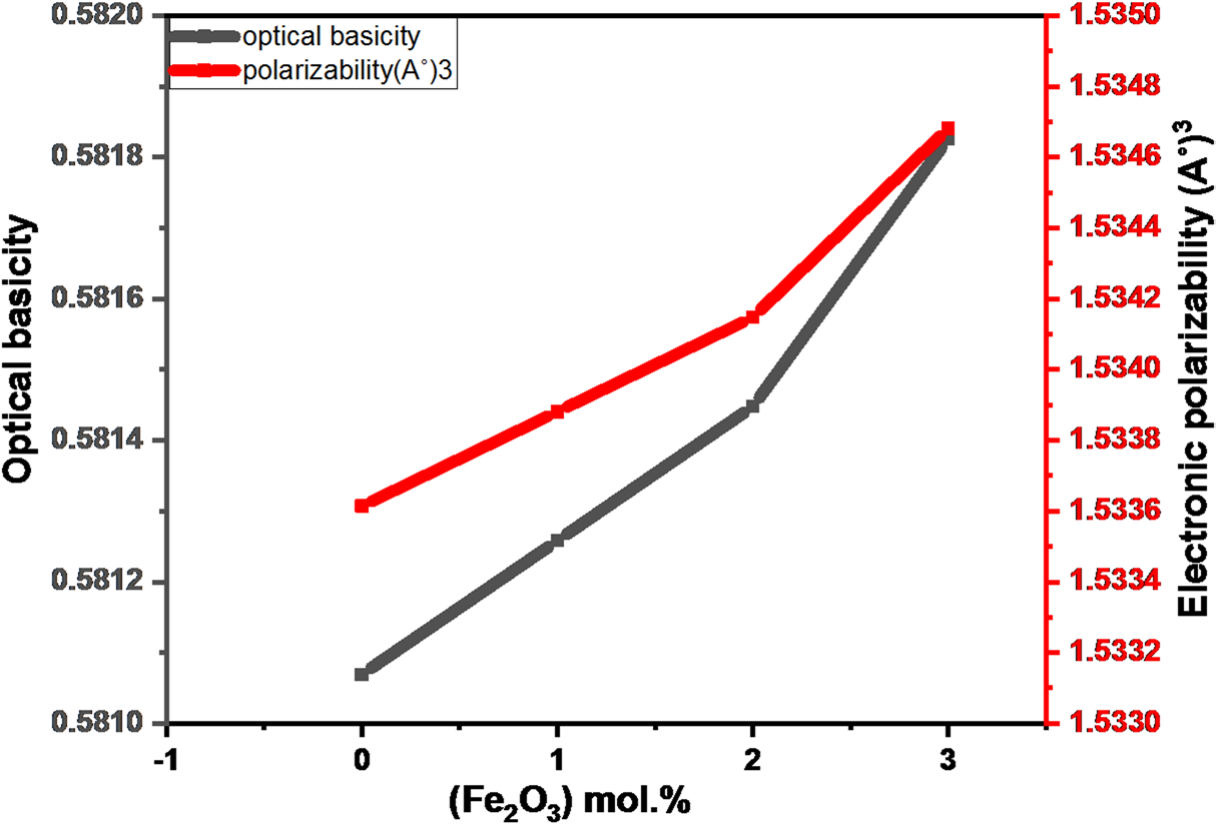
Variation of electronic polarizability and optical basicity versus with Fe_2_O_3_ mol% content.

The calculated values of $\:{ \Lambda }_{\mathrm{t}\mathrm{h}}$, $\:{{\upalpha\:}}_{0}^{-2}$ and$\:\:{{\upchi\:}}_{\mathrm{a}\mathrm{v}}$were noted in Table 6. Figure 12 presents the variation of the optical basicity ($\:{ \Lambda }_{\mathrm{t}\mathrm{h}}$) and the electronic polarizability of oxide ion ($\:{{\upalpha\:}}_{0}^{-2})$ versus the concentration of Fe_2_O_3_ in a system of Fe_2_O_3_ doped B_2_O_3_.CaO.P_2_O_5_.Na_2_O glasses. This increase in $\:{ \Lambda }_{\mathrm{t}\mathrm{h}}$ is due to increase in the average polarizability and increment in the refractive index^13,27^.

**Table 6:** The physical parameters of the glass samples.

Sample	Fe0	Fe0.5	Fe1	Fe2
Theoretical optical basicity	0.581	0.5812	0.5814	0.582
electronic Polarizability(A˚)^3^	1.533	1.5338	1.534	1.5347
Electronegativity	2.641	2.6403	2.6398	2.639
Metallization criterion	0.396	0.353	0.348	0.344
Experimental density (± 0.01)(g/cm^3^)	2.158	2.17	2.313	2.33
Empirical density(g/cm^3^)	2.616	2.63	2.64	2.67
Molar volume (± 0.2)(cm^3^/mol.)	32.49	32.53	30.72	30.84
Bandgap energy (eV)	3.138 ± 0.05	2.49 ± 0.03	2.42 ± 0.04	2.36 ± 0.05
Refractive index	2.36 ± 0.05	2.55 ± 0.03	2.574 ± 0.04	2.595 ± 0.05
Dielectric Constant(A˚)^3^	5.57	6.5	6.62	6.73
Molar refraction	19.62	21.81	21.75	24.615
Optical Dielectric Constant	4.574	5.5	5.62	5.73
The molar electronic polarizability(A˚)^3^	7.787	8.656	8.629	9.768

## Conclusion

The impact of varying α-Fe_2_O_3_NPs concentrations on the structural characteristics of the (68-x) B₂O₃.20CaO.2P₂O₅. 10Na₂O.xα-Fe₂O₃ (x = 0,0.5, 1, and 2 mol%) have illustrated in this investigation. For the current samples, both the optical and physical properties were examined. Mass density increases because the lighter molecular weight of boron oxide replaces the larger molecular weight of Fe_2_O₃. thus, the results are shown that the metallization M values vary between 0.39 and 0.34. As a result, the falling output indicates that the samples are semiconductors. The variations in the samples’ elastic properties responded to packing density changes in a comparable way. The elastic modulus decreased as a result of the glass sample’s ionic radii being raised by the addition of Fe_2_O_3_. where the ionic radii of the Fe^3+^ (0.92A^o^) are greater than those of the borate ion (0.351A^o^). In general, the borophosphate glass-ceramic system is an intriguing subject for materials science research, primarily due to its potential for diverse biomedical and technological applications. This composition, featuring the synergistic properties of a borophosphate matrix and varying concentrations of Fe_2_O_3_ offers a versatile platform. Furthermore, the iron oxide can influence the optical properties and increased the density of the material, suggesting potential for applications in radiation shielding.

## Data Availability

All data generated or analyzed during this study are included in the manuscript. Additional data or data files can be provided by the corresponding author upon request.
